# Antimicrobial Functionalization of Composite Nanofibrous Yarns as Surgical Sutures

**DOI:** 10.1002/mabi.202500510

**Published:** 2026-02-24

**Authors:** Věra Hedvičáková, Manikandan Sivan, Divyabharathi Madheswaran, Kristýna Havlíčková, Šárka Hauzerová, Maxim Lisnenko, Jan Valtera, Jaroslav Mikule, Kateřina Strnadová, Věra Jenčová, Eva Kuželová Košťáková, David Lukáš

**Affiliations:** ^1^ Department of Chemistry–Bioengineering Faculty of Science, Humanities, and Education Technical University of Liberec Liberec Czech Republic; ^2^ Department of Tissue engineering Institute of Experimental Medicine of The Czech Academy of Sciences Prague Czech Republic; ^3^ Department of Textile Machine Design Faculty of Mechanical Engineering Technical University of Liberec Liberec Czech Republic

**Keywords:** AC electrospinning, composite nanofibrous yarns, drug loading system

## Abstract

Antimicrobial drug‐releasing sutures have the potential to minimize the risk of postoperative inflammation and infection development. Using such medical devices is patient‐considerate and cost‐effective, reducing the need for oral drug administration and secondary surgical interventions. Nowadays, antimicrobial coatings on surgical sutures exist, however, they typically provide short‐term drug release with limited concentrations. In this study, alternating current electrospinning was utilized to produce pristine and chlorhexidine (CHX) loaded composite polycaprolactone nanofibrous yarns with a mechanically resistant polyamide 6 core. Production speed between 10 and 30 m/min resulted in varying linear densities of yarns inversely proportional to the production speed and consequently with different concentrations of CHX. A prolonged release lasting one month was achieved, attributed to the dual relaxation times. Morphological analyses showed a composite character of yarns with a uniform pristine or CHX‐loaded fibrous envelope that was susceptible to enzymatic degradation. The yarns exhibited high porosity, exceeding values typical for conventional fibers and displayed mechanical properties compatible with thin monofilaments sutures. The estimated curvature and torsion of the fibers, combined with the nanofibrous envelope resulted in a 3D yarn structure that closely mimics the extracellular environment. The 3D nature of composite nanofibrous yarns together with adsorbed proteins supported fibroblast adhesion and proliferation indicating biocompatibility. Proposed composite nanofibrous yarns represent an alternative to conventional smooth dip‐coated antimicrobial sutures.

## Graphical abstract

This study presents the fabrication of drug‐loaded nanofibrous yarns using alternating current electrospinning. Incorporating chlorhexidine into a polycaprolactone envelope around a polyamide 6 core, the yarns offer extended antimicrobial release and promote cell adhesion. The structure mimics natural tissue, suggesting potential as a long‐term, biocompatible alternative to traditional antimicrobial sutures.

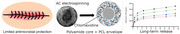

## 1 Introduction

Wounds and lacerations have been a major health risk without proper treatment. The necessity of wound closure has driven the development of a number of materials that have been used to approximate the injured tissues. Historically, animal tendons, plant fiber, steel or iron wires and later catgut were used. Nowadays, surgical sutures made from materials like surgical silk, polydioxanone or poly (lactic‐co‐glycolic acid) are used. Over time, additional requirements such as facilitating the healing without scarring, withstanding the tissue induced stress and offering flexibility of the suture material could be addressed through the emergence of a diverse suture material. Moreover, as postoperative surgical site infection (SSI) or inflammatory response may be the reaction of the body to suture implantation, biologically active systems may be a promising solution [1]. Therefore, antimicrobial sutures, serving as a local drug delivery system, reduce the risk of secondary operative interventions.

Currently, commercially available antimicrobial surgical sutures are produced using dip coating techniques as synthetic sutures are typically manufactured by melt extrusion [2, 3]. Surface modification is another option for suture functionalization. For both methods, there is a risk of fast release together with limited drug‐loading capacity, which is not suitable for long‐healing wounds [4]. To address these limitations, polymer‐drug blending offers a promising strategy to produce drug‐eluting surgical sutures [5].

In recent developments, composite nanofibrous sutures (CNSs), also known as core nanofibrous sheath yarns, have emerged as promising candidates for advanced suture materials [5, 6, 7]. These structures combine a microscale core, typically made of conventional yarn or filaments, with a surrounding nanofibrous envelope. The core component provides the mechanical strength required to maintain wound closure and resist tensile forces during the healing process. At the same time, the nanofibrous envelope resembles the natural extracellular matrix, supporting the cellular activities (adhesion, migration, and proliferation [8]. In addition, its high surface area and porosity allow for the incorporation of bioactive substances such as antimicrobial agents, anti‐inflammatory drugs, growth factors, and pain relievers, enhancing the therapeutic function of the suture and contributing to faster wound healing.

Creating core sheath yarns by coating microfilament cores with electrospun nanofibers is challenging when using conventional direct current (DC) electrospinning. DC systems require a grounded collector to attract the charged fibers, but due to mutual repulsion between like charged jets, the fibers tend to spread out, making uniform deposition on a nonconductive core yarn difficult. Additionally, the geometry and position of the collector significantly influence fiber deposition, adding further complexity to the process [9, 10].

In contrast, alternating current (AC) electrospinning offers a more practical and efficient approach. Under high AC voltage, the electric field alternates rapidly, inducing both positive and negative charges in the spinning solution [11]. This leads to the formation of a counter‐ionic region a few centimeters from the spinning electrode, often referred to as a virtual collector. In this region, the charges carried by the polymer jet undergo recombination, resulting in partial or complete neutralization. As a result, a dense and mechanically coherent fibrous cloud, known as a fibrous plume, is formed. Driven by an electric wind, this plume can be directed toward nonconductive or moving substrates such as core yarns, enabling continuous and uniform deposition of nanofibers and facilitating the scalable production of core nanofibrous sheath yarns [6].

This study aims to develop and characterize antimicrobial composite nanofibrous yarns, fabricated using AC electrospinning, for application in surgical sutures. The resultant composite yarns consisted of a polyamide 6 (PA6) core yarn covered with a poly‐ε‐caprolactone (PCL) nanofibrous envelope. Although PA6 is a non‐resorbable polymer—unlike PCL, which is fully biodegradable—it was intentionally selected as the core material in order to provide a stable, inert mechanical support. This design choice allows the present study to focus specifically on the behavior, structure, and functionality of the resorbable PCL envelope without the confounding effects of a degrading core. Moreover, as AC electrospinning is performed at room temperature, the PCL envelope was produced from a polymeric blend with the antimicrobial agent chlorhexidine (CHX) to enable sustained drug release. Variability in surgical sutures is desired as no suture material can meet all clinical needs. To achieve this, various production speeds ranging from 10–30 m/min were used resulting in composite nanofibrous yarns with a range of linear densities and varying CHX concentrations.

## 2 Results and Discussion

Material selection is a crucial step in the design of surgical sutures. In this study, we developed a composite nanofibrous yarns consisting of PA6 core yarn with nanofibrous PCL envelope, a biocompatible, non‐toxic polymer approved by the Food and Drug Administration (FDA).

### 2.1 Morphology of Composite Nanofibrous Yarn

The composite nanofibrous yarns were fabricated using AC electrospinning. The PA6 core yarn provides the necessary mechanical strength to the composite yarn. PCL nanofibrous envelope does not withstand the mechanical demands typically applied on surgical sutures. However, focus on the interface between the surgical suture and the tissue is important in order to secure the appropriate healing process completed by the formation of mechanically resistant tissue.

Pristine and drug‐loaded composite nanofibrous yarns were fabricated with various production rates using AC electrospinning (10, 15, 20, 25, and 30 m/min, sample naming of pristine yarns: PCL_10, PCL_15, PCL_20, PCL_25, and PCL_30 and sample naming of CHX‐loaded yarns: PCL+CHX_10, PCL+CHX_15, PCL+CHX_20, PCL+CHX_25, and PCL+CHX_30). The linear density of the resultant yarns was directly influenced by the production speed of the core yarn, or in other words, the winding speed or withdrawal rate. Thanks to the applied AC potential, which makes an electrically neutral compact fibrous plume, the compact fibrous plume further moved towards the ballooning core yarn with the help of corona wind. Due to the sticky nature of the fibrous plume, it was wrapped around the core yarn.

As previously discussed, AC spinning overcomes the limitations of dip coating and surface drug immobilization. In contrast, the melt extrusion process is a solvent‐free method suitable for poorly soluble drugs. However, these issues are not associated with CHX/PCL blend spinning. CHX is an antimicrobial agent targeting the anionic lipids in membranes and is active against Gram‐positive and Gram‐negative bacteria and fungi [12, 13]. Currently, commercially available antimicrobial sutures are mainly produced based on the triclosan coating to prevent the development of SSIs [14, 15]. However, there are concerns about spreading the triclosan antibiotic resistance as triclosan is also widely used for commercial hygiene and disinfection purposes [16]. Therefore, in this study CHX was selected as an alternative to triclosan.

**FIGURE 1:**
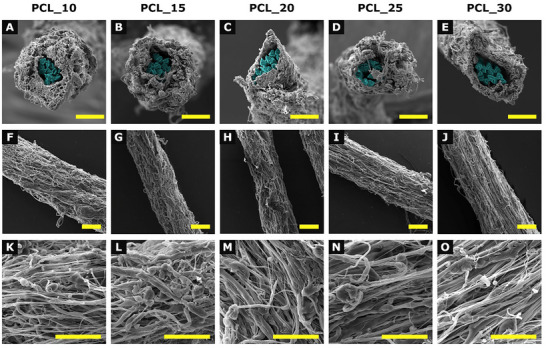
Scanning electron microscopy micrographs of pristine composite nanofibrous yarns prepared at different production speeds. Panels (A–E) show cross‐sections at 1000× magnification, with PA6 highlighted in blue, (F–J) present longitudinal views at 500×, and (K–O) display surface morphology details at 3000×. Columns correspond to individual sample groups: A/F/K: PCL _10, B/G/L: PCL _15, C/H/M: PCL_20, D/I/N: PCL _25, and E/J/O: PCL _30. Scale bar: 40 µm.

**FIGURE 2:**
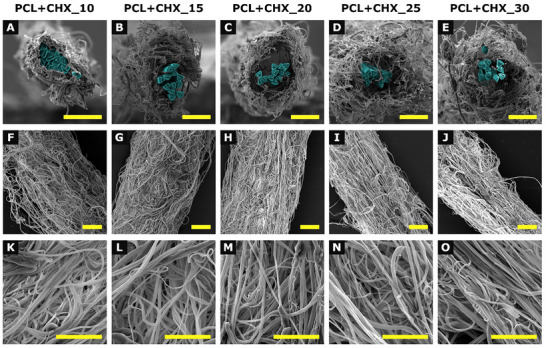
Scanning electron microscopy micrographs of CHX‐loaded composite nanofibrous yarns prepared at different production speeds. Panels (A–E) show cross‐sections at 1000× magnification, with PA6 highlighted in blue, (F–J) present longitudinal views at 500×, and (K–O) display surface morphology details at 3000×. Columns correspond to individual sample groups: A/F/K: PCL+CHX _10, B/G/L: PCL+CHX _15, C/H/M: PCL_20+CHX, D/I/N: PCL+CHX _25, and E/J/O: PCL+CHX _30. Scale bar: 40 µm.

The surface morphology of the resultant yarns is shown in Figures 1 and 2. The yarns exhibit a uniformly coated electrospun fibrous morphology, with cross‐sectional images revealing a composite structure of a nanofibrous PCL envelope coated on the PA6 core yarn. The pristine nanofibrous envelope displayed typical “beads‐on‐string” morphology. In contrast, the CHX‐loaded yarns showed smooth, bead‐free fibers.

CHX may act like a surfactant by modifying solvent‐polymer interactions and reducing surface tension. While CHX lowers the viscosity of the PCL solution, it significantly increases solution conductivity and charge density. This increase strengthens the electrostatic forces stretching the jet during electrospinning, stabilizing it and preventing bead formation by suppressing the Plateau–Rayleigh instability. Thus, despite the lower viscosity, the jet remains stable and produces smooth, bead‐free fibers in the presence of CHX [17, 18].

**FIGURE 3:**
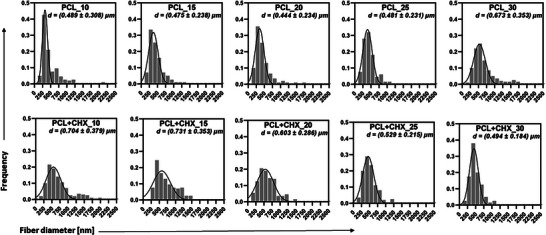
Histograms of various pristine and CHX‐loaded composite nanofibrous yarns and fiber diameters.

**FIGURE 4:**
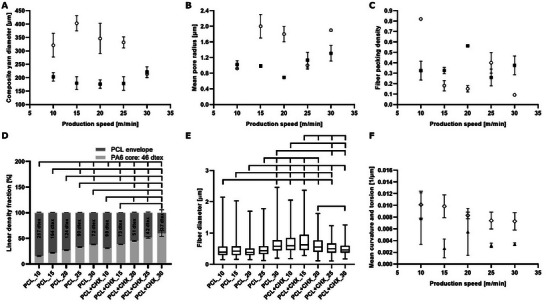
(A) Diameter of composite nanofibrous yarns as a function of production rate for pristine (black squares) and CHX‐loaded (white circles) samples. Significant differences were observed for the following comparisons: PCL_10 > PCL+CHX_10, PCL+CHX_15; PCL_15 and PCL_20 > PCL_30 and PCL+CHX_10, PCL+CHX_15, PCL+CHX_20, PCL+CHX_25; PCL_25 > PCL+CHX_10, PCL+CHX_15, PCL+CHX_20, PCL+CHX_25. (B) The dependence of the mean pore radius of nanofibrous envelope of the composite nanofibrous yarns on the production rate is plotted for pristine (black squares) and CHX‐loaded (white circles) samples. (C) The dependence of the fiber packing density of nanofibrous envelope of the composite nanofibrous yarns on the production rate is plotted for pristine (black squares) and CHX‐loaded (white circles) samples. (D) Linear density fraction of core yarns and nanofibrous envelopes, weight % of various pristine and CHX‐loaded composite nanofibrous yarns. Statistical significance is denoted above the columns (*p* < 0.05). (E) The dependence of the fiber diameter in nanofibrous envelopes of the composite nanofibrous yarns on the production rate is plotted for pristine (black squares) and CHX‐loaded (white circles) samples. (F) Dependence of mean curvature (white rhombus) and torsion (black triangle) of nanofibers on the surface of the pristine composite nanofibrous yarn.

Figure 3 shows detailed histograms of fiber diameters divided into bins of equal width of 125 nm. The composite yarn diameter was measured from low‐magnification scanning electron microscopy (SEM) images on 25 randomly selected sections of composite yarns. The results of the yarn diameter analysis are shown in Figure 4A. The average yarn diameters range from 150 to 400 µm. The CHX‐loaded yarns generally exhibit greater thickness than those of the pristine nanofibrous composite yarns. The dependence on the production rate is not insignificant for both types of samples. Most probable pore radii (Figure 4B) are between 1 and 2 µm, which is extraordinary compared to conventional fibers1. As production speed increases, the mean pore radius tends to increase for CHX‐loaded yarns but remains relatively constant for pristine samples [19, 20]. This suggests that CHX addition facilitates structural changes at higher spinning speeds, possibly due to enhanced charge density and altered jet dynamics, leading to looser fiber packing and larger pore sizes. Correspondingly, fiber packing density (Figure 4C), typically ranges between 0.3 and 0.4, corresponding to sheath porosities of 0.6–0.7, decreases at high speeds for both formulations, with the effect being more pronounced in CHX‐loaded samples.

The linear density of the resultant yarns was analyzed using the gravimetric method. As shown in Figure 4D, the linear density of the pristine and CHX‐loaded composite nanofibrous yarns was mainly influenced by the production speed of the core yarn. The linear density of the resultant yarn is inversely proportional to the production speed of the core yarn. If the production speed of the yarn is low, a greater amount of nanofibers is deposited on the core yarn. In contrast, if the core yarn production speed is higher, a smaller amount of nanofibers is coated on the core yarn. The linear density of the pristine suture is higher than the CHX‐loaded suture. This could be attributed to the fact that the addition of CHX into the spinning solution resulted in a lower production rate.

The average values of nanofiber diameters are not significantly dependent on the production rate and are also not significantly affected by CHX incorporation, as shown in Figure 4E.

Current microscopic techniques do not allow us to observe the arrangement of nanofibrous fibrils inside nanofibrous yarns produced by AC electrospinning. However, their structural parameters/features/properties can be roughly estimated from their surface layers. The average values of curvature and torsion are determined using a procedure proposed by DeHoff [21]. Apart from a single deviating torsion measurement for a production speed of 15 m/min, the values of average curvature and torsion (Figure 4F) show a weakly decreasing character with an increase in production speed. This increase of nanofibrous plume stretching can be explained by the more intensive elongation of the nanofiber plume with increasing production speed.

Using Equation (6) and the imaging of the corresponding helixes, we can compare typical nanofiber shapes for the extreme values of production rates, i.e., 10 and 30 m/min. For a production velocity of 10 m/min, Equation (6) give the radius of the helix $a=\frac{0.0101}{0.0101^{2}+0.0109^{2}}\;\mu \;m^{-1}=45.7\;\mu m^{-1}$ and the pitch of the value $2\pi b=2\pi \frac{0.0109}{0.0101^{2}+0.0109^{2}}\mu \;m^{-1}=310.1\;\mu m^{-1}$. For a production speed of 30 m/min, we obtain the radius of the helix $a=\frac{0.00835}{0.00835^{2}+0.00353^{2}}\;\mu \;m^{-1}=101.6\;\mu m^{-1}$ and the pitch of $2\pi b=2\pi \frac{0.00353}{0.00835^{2}+0.00353^{2}}\mu \;m^{-1}=269.8\;\mu m^{-1}$. Both helices are shown in Figure S1A to give an idea of the curl of surface fibers in a nanofiber envelope of composite nanofiber yarns.

The advantage of the composite nanofibrous yarns lies in the fact that the nanofibrous envelope around the PA6 core yarn contains significantly more fibers than the PA6 core yarn itself. This fibrous structure closely mimics the filamentous nature of the natural cellular environment, extracellular matrix. The number of nanofibers per cross section was determined for a selected representative sample of the composite nanofibrous yarn, PCL+CHX_20. For this sample the following values were measured: average cross‐sectional fiber density of the composite nanofibrous yarn N_A_ = 0.0197 µm^−2^, average thickness of the nanofibrous envelope d = 38±9 µm, total number of fibers per cross‐section of the nanofibrous envelope N = 507 ± 120.

### 2.2 Mechanical Testing

Mechanical testing was performed only for the PCL envelopes produced at the lowest and highest production speeds, both in pristine and CHX‐loaded composite nanofibrous yarns, together with the PA6 core yarn serving as a reference (Table 1). The composite nanofibrous yarns were pliable and easy to handle. PCL+CHX_30 exhibited the highest tensile strength (Figure S2A). This likely reflects fiber alignment at higher production speed, further enhanced by the CHX‐induced reduction in bead density. PA6 showed higher Young's modulus compared to PCL+CHX_30 (Figure S2B) and PCL_10 showed higher modulus compared to PCL_30, PCL+CHX_30 where the stiffness might be reduced by increasing fiber alignment and lowering inter‐fiber entanglement. The further decrease in modulus for PCL+CHX_10 likely reflects CHX‐induced disruption of PCL crystallinity, an effect most evident at low production speed. Elongation at break (Figure S2C) was highest for CHX‐loaded samples, likely due to CHX‐induced reduction of crystallinity and improved fibre uniformity.

**TABLE 1:** Summary of mechanical properties of the composite nanofibrous yarns PCL_10, PCL_30, PCL + CHX_10, PCL + CHX_30 and PA6 core. For tensile strength, Young's modulus, elongation at break, and knot performance the values are presented as mean ± SD. The knotted‐to‐unknotted strength ratio reflects the percentage of residual strength retained after knotting.

Parameter	PCL_10	PCL_30	PCL + CHX_10	PCL + CHX_30	PA6
Tensile Strength [MPa]	443.07 ± 23.73	440 ± 35.65	442.39 ± 15.85	484.37 ± 26.41	447.17 ± 8.39
Young's Modulus [GPa]	1.22 ± 0.09	0.93 ± 0.09	1.1 ± 0.09	1.03 ± 0.13	1.15 ± 0.12
Elongation at Break [%]	39 ± 1.8	44.88 ± 3.87	53.21 ± 3.41	54.49 ± 2.98	40.89 ± 1.96
Knot Pull Strength [N]	1.72 ± 0.16	1.51 ± 0.22	1.63 ± 0.18	1.46 ± 0.34	1.43 ± 0.25
Knotted‐to‐unknotted strength ratio	96.6%	85.3%	91.6%	74.9%	79.4%

Knot pull strength (Figure S2D) did not differ significantly among the groups, demonstrating that variations in processing parameters and CHX loading do not compromise this clinically important mechanical parameter. The knot appeared stable with minimal tendency to slip. The knotted‐to‐unknotted strength ratio reflected the overall mechanical trends: PCL_10 showed the smallest strength loss upon knotting (∼97%), whereas higher production speed and CHX addition led to lower ratios (down to ∼75% for PCL+CHX_30). This decrease corresponds to the reduced stiffness and higher ductility. PA6 showed an intermediate ratio (∼80%), consistent with its mechanical profile.

Nonabsorbable sutures with diameters of 100–149 µm exhibit tensile strengths of 6.57 N for silk and 3.25 N for polyamide, with corresponding knot resistance values of 0.31 and 3 N, respectively [22]. In thicker polyamide filaments (150–190 µm), combining knot configurations—such as a square knot followed by a surgeon's knot—further increases knot tensile resistance to approximately 20 N. Elongation at break for nylon sutures of comparable diameters ranges from roughly 80%–97% [23]. For polypropylene, the failure load in knot varies substantially with diameter, reaching 38.2 N for 420–440 µm monofilaments and approximately 3.5 N for 100 µm fibers [24]. Direct comparison with the composite nanofibrous yarns produced in this study is challenging, as the material architecture combines a nonabsorbable PA6 core with an absorbable PCL envelope with diameters ranging from 150 to 400 µm, with a multifilament PA6 core comprising 12 filaments and having an approximate diameter of 150 µm. Nevertheless, braiding of these composite nanofibrous yarns is expected to further enhance their load‐bearing behavior and knot security, allowing them to approach the performance characteristics typical of braided surgical sutures.

### 2.3 Release of Chlorhexidine

Drug eluting sutures are available on the market, however long‐term maintenance of effective concentrations poses an ongoing challenge [25].

**FIGURE 5:**
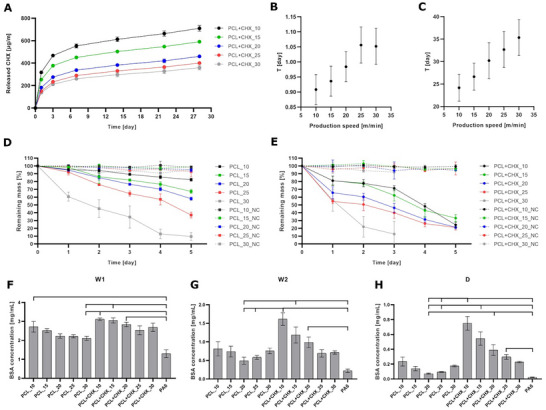
(A) Cumulative release of CHX from composite nanofibrous yarns prepared with various production rates. Relaxation time values (in days) as a function of production speed (m/min) for (B) shorter relaxation times and (C) longer relaxation times. Evolution of weight loss due to enzyme‐catalyzed degradation of (D) PCL envelopes and (E) CHX‐loaded PCL envelopes by lipase (2.5 U/mL) for 5 days. (F) Analysis of the interaction of capillary‐bound proteins after first wash (W1) in phosphate buffer saline (PBS), (G) weakly bound proteins in second wash (W2) in PBS and (H) strongly bound proteins released into the SDS desorption solution (D). Statistical significance is denoted above the columns (p < 0.05).

In this study, we address this limitation by even distribution of the CHX within the fibrous nature of the PCL envelope in the composite nanofibrous yarns. To confirm successful drug loading into nanofibrous PCL envelopes, release of CHX from drug‐loaded composite nanofibrous yarns was detected based on UV–vis spectroscopy (UV–vis) (Figure 5A). All tested samples demonstrated sustained CHX release for up to one month. Released concentrations of CHX were inversely proportional to the winding speeds indicating statistically higher drug loading capacity per unitary yarn length at lower production rates.

A model of first‐order release kinetics is used to describe the drug release into the medium. It is described by the Equation (1)

(1) $$\;\frac{dc}{dt}=-kc=-\frac{c}{\tau }$$

where *c(t)* is the drug concentration in the medium, *k* is the first‐order release constant and *τ* is the relaxation or characteristic time of release. This model can be used to describe the release of water‐soluble drugs in porous matrices [26].

The solution of Equation (1) can be written in the form

(2) $$c=A\;exp\left(-\frac{t}{\tau }\right)$$

However, Equation (2) does not provide a satisfactory regression for CHX release from composite nanofibrous yarns as the release kinetics exhibited an initial burst release followed by a slower and sustained release of the active substance supporting the potential for long term releasing yarns.

Therefore, it is possible to hypothesize that CHX release is controlled by two different mechanisms, e.g. CHX release from the surface of the fibers and release from their bulk, which is slower. In this case, the release is modelled by a function of

(3) $$c=A\;exp\left(-\frac{t}{\tau }\right)+B\;exp\left(-\frac{t}{T}\right)$$

with two relaxation times τ *a* *T*. Figure S1B–F shows shorter and longer relaxation times.

Interestingly, all data for different production rates behave similarly. The shorter relaxation time (Figure 5B) is approximately 1 day and the longer one varies from 24 to 35 days (Figure 5C).

Manufacturer‐stated CHX concentrations in marketed sutures are generally not disclosed. Obermeier et al. showed that CHX‐coated sutures released the drug continuously for up to 96 h after an initial burst, whereas commercial triclosan‐coated suture Vicryl Plus contain up to 472 µg/m of triclosan and release ∼69% within the first 24 h and nearly completely by 36 days [27]. Another study using CHX‐coated sutures with drug loadings of 1100, 2200, and 3300 µg/m demonstrated significant reduction of viable S. aureus on the suture surface compared to uncoated or triclosan‐coated sutures [28]. In contrast, Golling et al. reported that sutures coated with < 60 µg/m of CHX, a relatively low concentration, did not achieve a statistically significant reduction in infection [29]. Experimental approaches using very high CHX concentrations (e.g., 20% CHX digluconate, ∼200 mg/mL) in melt‐soaked monofilaments failed to provide reliable antibacterial protection, emphasizing that drug delivery method and fiber architecture are critical [30].

In our previous work on composite nanofibrous yarns produced by AC spinning with antibacterial nanofibrous envelope doped with CHX, designed for suture applications, we experimentally demonstrated effective antibacterial activity of drug‐loaded composite nanofibrous yarn‐based structures against clinically relevant bacterial strains [7]. Although the material differs from the present system, these results support the relevance of nanofibrous yarn architecture as promising platforms for antibacterial suture development. Based on our calculations, the prepared composite nanofibrous yarns function as long‐term drug delivery systems with two distinct relaxation times: an initial burst release followed by a prolonged release phase lasting up to one month, with CHX release reaching ∼360 µg/m for PCL+CHX_30 and up to ∼710 µg/m for PCL+CHX_10. Taken together, these comparisons suggest that our CHX‐loaded nanofibrous yarns, with controlled drug loading and sustained release from a 3D nanofibrous envelope, represent a promising platform for antibacterial suture development. They may offer advantages over some existing commercial CHX or triclosan sutures in terms of drug delivery kinetics, while also providing favorable mechanical and biocompatible properties. Further in vitro and in vivo studies are warranted to quantitatively benchmark performance against marketed products. Although antibacterial evaluation is necessary to fully validate the performance of these CHX‐loaded structures, such testing was beyond the scope of this study. The prepared composite nanofibrous yarns are intended to undergo subsequent braiding, which will protect the nanofibrous sheath and enable further adjustment of the final CHX loading. By modifying all or only selected filaments prior to braiding, the resulting sutures can achieve CHX concentrations sufficient for effective antibacterial inhibition.

### 2.4 Accelerated Degradation

In this study, a non‐absorbable PA6 core yarn was intentionally employed to provide stable, non‐degrading support, allowing us to isolate and monitor the enzyme‐catalyzed hydrolytic degradation of the PCL envelope. Although the composite yarn therefore contains a non‐absorbable component, our long‐term aim is to translate this concept toward fully absorbable suture systems, which are clinically preferred for many applications because they gradually degrade and do not require removal. Nevertheless, non‐absorbable sutures remain widely used, and their favorable handling characteristics and knot security make PA6 core yarn a suitable model core material for the controlled evaluation performed in this study [31]. From the enzyme‐catalyzed degradation results, the effect of winding speed on the formation of the nanofibrous yarn envelope is clearly evident for either pristine or drug‐loaded composite nanofibrous yarns. As the electrospinning rate increases, faster degradation of the PCL envelope and more significant weight loss can be observed on each test day compared to the negative control (NC, without lipase), (Figure 5D,E; Figure S3B–F). The presented degradation occurs with an excess of substrate. Degradation occurs on the surface of the fibers, the presence/penetration of water and enzyme is necessary [32, 33]. Since PCL is hydrophobic, water and enzymes will have difficulty penetrating the internal structure of the nanofiber layer and thus gradually degrade the fibers currently present on the surface of the layer. In less dense layers (those with slower production speed), the substrate is more accessible to the enzyme, in thicker layers the enzyme reaches the substrate more slowly—gradually.

This is also related to the fact that the enzymes act preferentially on the surface of the fibrous material [32, 33]. Therefore, keeping the same concentration of enzyme (2.5 U/mL) and the same length of the tested composite yarns (1 m), i.e. comparable surface area, we observe more significant degradation effects in samples produced at higher production speed, i.e. in samples with lower PCL packing concentration, because the enzyme acts on a lower amount of substrate.

By comparing pristine composite yarns with a PCL envelope and drug‐loaded composite yarns with a CHX‐loaded PCL envelope at individual degradation time points, it is possible to observe the influence of the incorporated CHX. Composite yarns with a CHX‐loaded envelope show different yarn finesses compared to composite yarns with a PCL envelope, while maintaining the same process conditions. CHX‐loaded PCL envelopes applied to the yarn core achieve significantly lower weights (by 55% lower) compared to PCL envelopes. The presence of CHX probably affects the spinnability of solutions, or rather the productivity of the nanofibrous envelope application. The nanofibrous envelopes with CHX are bulkier (“fluffier”) and more porous, and for most production speeds we observe higher mean pore sizes (Figure 4B) in the PCL nanofibrous envelope. This enables faster and deeper penetration of water into the nanofibrous structure, which significantly promotes bulk degradation of the PCL nanofibrous envelope [34]. Literature shows that the porosity of the material and the rate of water penetration into its structure significantly accelerate the degradation process [35, 36, 37]. At the same time, for CHX‐doped PCL nanofibrous envelopes, at nearly all production speeds, we observed lower fiber packing density (Figure 4C), which supports the macroscopic observation of a bulkier structure in composite yarns with CHX‐doped PCL envelopes. Larger pores and decreased fiber packing density are both factors that enhance mass transport and enzyme accessibility within the envelope structure, thereby promoting more rapid degradation [38]. This leads to faster hydrolytic cleavage of ester bonds in PCL chains, because PCL degradation is diffusion‐limited, that is, the degradation rate is governed by water accessibility [39, 40]. Simultaneously, the release of CHX molecules from the doped nanofibrous envelopes likely causes the formation of local cavities, thereby increasing the porosity of the nanofibrous material [41]. Moreover, incorporation of additives such as CHX in this study may disrupt the crystalline structure of the polymer, leading to reduced crystallinity and accelerated degradation rates. Small molecules such as CHX may also interfere with the tight packing of polymer chains and increase the microheterogeneity of the matrix [42].

Together, these findings demonstrate that production speed and CHX modulate core‐sheath yarn microstructure via physically relevant parameters such as pore size and packing density, which in turn accelerate degradation kinetics. This is observed not only in higher weight losses (Figure 5E), but also in more significant morphological changes of nanofibers compared to yarns with undoped PCL envelope. For CHX‐loaded envelopes, we observed more significant fiber restructuring, the formation of defects and the breakdown of the fiber structure (Figure S3A). At the same time, we again observed the influence of the concentration (weight) of the substrate that is degraded. Under identical conditions, it is possible to observe a trend that the lower the weight of the CHX‐loaded PCL envelope, the faster the envelope is degraded (Figure 5E). These structure‐property relationships are critical for designing tunable fibers with tailored degradation appropriate for biomedical applications.

### 2.5 Protein Adsorption

Unlike natural materials, synthetic ones do not offer specific receptor binding sites for cell adhesion molecules. As a result, cell adhesion on synthetic materials is guided by non‐specific interactions, primarily governed by the adsorption of proteins that precedes cell adhesion. The dynamics of this protein adsorption are driven by surface chemistry that is affected by charge, hydrophobicity, and topography [43]. These dynamics, in turn modulate cell adhesion. In this study, we used a synthetic, hydrophobic PCL nanofibrous envelope, for which protein adsorption was quantified.

As part of testing the interaction of proteins with pristine and drug‐loaded composite nanofibrous yarns, a number of capillary‐bound, weakly and strongly bound proteins were analyzed. Capillary‐bound proteins interact with proteins bound by weak and strong interactions to the surface of nanofibers, only by very weak interactions. In the structure of the material, proteins are bound due to capillary forces caused by the fibrous structure of the material. Capillary‐bound proteins are released from the material by the first wash with PBS (proteins marked as W1). Weakly bound proteins interact by weak non‐binding interactions with proteins bound to the surface of the fibers. These proteins are released by the second wash with PBS (proteins marked as W2). Proteins that interact with the surface of the fibers and proteins that are bound to this layer by strong non‐binding interactions cannot be released by simply rinsing in PBS. These proteins are strongly bound and are released from the material by the action of a desorption solution (proteins marked as D) [44].

The results show that with increasing core yarn withdrawal rate during the spinning process, and therefore decreasing nanofibrous layer thickness, the number of adsorbed proteins decreases. This trend is observable both for pristine (PCL) and for drug‐loaded yarns (PCL+CHX). For capillary‐bound proteins (Figure 5F), there is no significant difference in the number of bound proteins on pristine yarns and on drug‐loaded yarns produced with the same withdrawal rate. For weakly and strongly bound proteins (Figure 5G,H), the effect of incorporated CHX on the number of bound proteins is evident. This may be due to the resulting structure of the nanofibrous envelope. CHX‐loaded PCL envelopes are more porous (“fluffier”) compared to the morphology of the pristine PCL envelope. Moreover, since CHX contains numerous amino groups, proteins can easily adsorb onto the material through non‐covalent interaction [45]. The loose fibrous structure of the drug‐loaded yarns probably allows for higher liquid permeability through the given material and thus a larger active surface for the possibility of protein adsorption. The overall protein adsorbed (Figure S4A) shows the same trend as described for W1, W2 and D individually.

For the qualitative analysis of adsorbed proteins, sodium dodecyl sulfate–polyacrylamide gel electrophoresis (SDS‐PAGE) analysis was used (Figure S4B–E). In each gel, samples of capillary‐bound proteins (W1), weakly bound (W2) and strongly bound (D) proteins from the tested materials were separated. The SDS‐PAGE results correspond to the results of protein quantification (Figure 5F–H).

### 2.6 In Vitro Testing of Non‐Functionalized Yarns

Low tissue reactivity shortens healing time and reduces scar tissue formation. It is not only necessary for proper tissue mechanics, but it is also an aesthetic factor. In this study, the composite nanofibrous yarns were covered with a nanofibrous PCL envelope that as opposed to extruded smooth filaments mimic the fibrous nature of the extracellular matrix. The specific surface area of the nanofibers offers numerous contact points for cell adhesion. Additionally, the 3D nature of the nanofibers offers non‐specific stimuli for adhered cells that can trigger cell proliferation and direct the proper cell fate [46].

**FIGURE 6:**
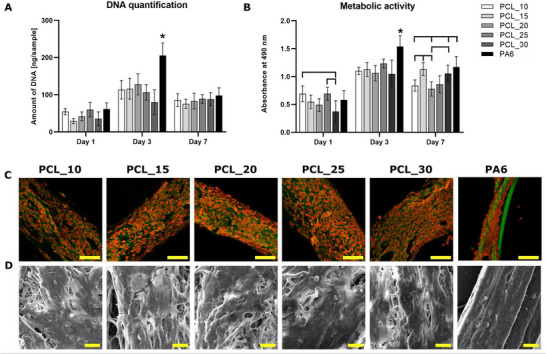
NIH‐3T3 fibroblasts seeded on the non‐functionalized composite nanofibrous yarns and PA6 core yarn. (A) DNA quantification measured by dsDNA assay kit and (B) metabolic activity, measured by MTS assay. Statistical significance is denoted above the columns (*p* < 0.05), * means the statistically highest value on the tested day. Visualization of NIH‐3T3 fibroblasts adhesion and distribution using (C) confocal microscopy on day 3, cell membranes were stained by DiOC6(3) (green color), cell nuclei by propidium iodide (red color), scale bar 100 µm. (D) Scanning electron microscopy on day 7, magnification 3000×, scale bar 20 µm.

To evaluate the effect of nanofibrous PCL envelopes in composite yarns compared to the PA6 core yarn, the pristine composite nanofibrous yarns were seeded with NIH‐3T3 fibroblasts. As the yarns seeding method allows the cell adhesion on the yarns using a small volume of culture medium, only pristine composite nanofibrous yarns, without CHX, were tested. DNA quantification of adhered cells is shown in Figure 6A. On day 1, uniform cell seeding was observed across all tested groups. On day 3, DNA content had increased in all samples, with the PA6 core yarn showing statistically highest content of DNA. On day 7, DNA levels decreased across all samples. Metabolic activity measurement (Figure 6B) revealed on day 1 statistically lower activity on PCL_30 compared to PCL_10 and PCL_25. On day 3, the highest metabolic activity was detected on PA6 core yarn which reflects the highest amount of DNA detected on this sample by DNA quantification. On day 7, cells on PCL_15, PCL_30, and PA6 core yarn displayed higher metabolic activity compared to other tested groups.

Confocal microscopy (Figure 6C) and SEM visualization (Figure 6D) showed that cells exhibited the spindle‐shaped morphology typical of well‐spread and adhered fibroblasts on all tested pristine yarns. By day 3, confluent layers of cells with oval‐shaped nuclei were observed across all materials. This confluency likely triggered contact inhibition and could lead to cell peeling, therefore decrease in DNA content was detected on day 7. All these methods indicate that the composite nanofibrous yarns possess good biocompatibility and support cell adhesion.

## 3 Conclusion

Antimicrobial sutures could prevent suture‐associated SSIs. However, based on the physio‐chemical properties of the drugs and polymers, the selected suture manufacturing process can be limiting. In this study, AC electrospinning, a low‐temperature technique, was used for the continuous fabrication of composite nanofibrous yarns from a blend for application as a surgical suture. The process produced drug‐eluting yarns with a bioactive agent (CHX) evenly distributed within the PCL nanofibrous envelope surrounding a mechanically resistant PA6 core yarn. The choice of non‐absorbable PA6 core yarn was deliberate, as its mechanically stable and non‐degrading nature enabled us to isolate and evaluate the degradation behavior and drug‐release characteristics of the absorbable PCL envelope without interference from a degrading core. The pristine nanofibrous envelopes exhibited beads‐on‐a‐string morphology, whereas the CHX‐loaded fibers were smooth. However, the addition of CHX reduced the production rate. Unlike the smooth PA6 core yarn, the fibrous envelope of the composite nanofibrous yarns exhibit curvature and torsion, along with a higher density of nanofiber fibrils in the cross‐section, contributing to the yarns’ 3D nature. Despite minor production speed‐dependent variations in CHX‐loaded samples, the yarns consistently exhibited highly porous fibrous envelopes with most probable pore radii of 1–2 µm, markedly exceeding the porosity typical of conventional fibers. The combination of a mechanically stable PA6 core and a resorbable, PCL envelope yielded a semi‐resorbable composite nanofibrous yarn with measurable tensile strength, Young's modulus, elongation at break, and knot‐pull performance. Although direct comparison with commercial sutures is limited by differences in material composition, filament architecture, and overall diameter, the obtained mechanical performance indicates that the composite structure provides sufficient robustness for further development while offering added antimicrobial and biological functionality. The CHX release was controlled by two mechanisms, release from the surface of the fibers and release from their bulk, therefore sustained release of CHX lasting for up to one month was observed. Moreover, higher concentrations of CHX were released at lower winding speeds. The bulkier nature of CHX‐loaded envelopes led to significantly faster enzymatic degradation. Additionally, protein adsorption was found to be inversely proportional to winding speed, slower winding speed resulted in increased concentrations of adsorbed proteins.

An ideal suture material should support, not hinder, cell growth or the organization of connective tissue. The nanofibrous envelope demonstrated biocompatibility, promoting protein adsorption followed by cell adhesion and proliferation of NIH‐3T3 fibroblasts. Unlike smooth surfaces, the rough surface of nanofibers is nonspecific biomechanical stimuli that is favored with regard to suture‐tissue interface. Therefore, the interface between the biocompatible suture material and the surrounding tissue could promote better integration avoiding tissue rejection or scar tissue formation that could impact the final aesthetic result as well as shorten the healing time. Building on these findings, a future perspective of this work involves replacing the PA6 core yarn with a fully absorbable alternative, enabling the development of completely absorbable composite nanofibrous sutures suitable for broader clinical applications. Moreover, building on this release behavior, future work will focus on developing braided sutures in which CHX loading is controlled by modifying all or only selected filaments prior to braiding to achieve effective bacterial inhibition.

## 4 Methods

### 4.1 Composite Nanofibrous Yarns Preparation and Functionalization

#### 4.1.1 Materials

Polycaprolactone (PCL) (Mn = 80 000 g/mol) and chlorhexidine (CHX) (≥99.5%) were purchased from Sigma‐Aldrich. Acetic acid (99%), formic acid (98%), and acetone were obtained from PENTA. PA6 multifilament yarn (to act as core yarn in this study) with a linear density of 46 dtex/12 filaments were provided by Odetka s.r.o. (Vrbno pod Pradědem, Czech Republic).

#### 4.1.2 Preparation of Pristine and Drug‐Loaded Spinning Solution

The spinning solution was prepared by dissolving PCL pellets in a mixture of acetic acid/formic acid/acetone (1/1/1 v/v) so as to attain 10 wt.%. To prepare a drug‐loaded solution, 0.5 wt.% CHX, and 10 wt.% PCL were dissolved in a mixture of acetic acid/formic acid/acetone (1/1/1 v/v), the so‐called PCL‐PCL‐CHX. All solutions were stirred overnight at room temperature (RT) until a homogeneous solution was obtained.

#### 4.1.3 Fabrication of Pristine and Drug‐Loaded Composite Nanofibrous Yarns

The fabrication process of composite nanofibrous yarns was illustrated in our previous study [7]. A custom‐built AC electrospinning setup was employed, comprising a rod‐shaped spinneret (10 cm in length, 2 cm diameter at the head) integrated into a polymer solution reservoir. The solution was delivered to the spinneret head through a coaxial channel using a screw pump developed at the Technical University of Liberec (Czech Republic). The screw pump operated at 500 RPM, supplying the polymer solution at a flow rate of approximately 18 mL/min to ensure the formation of a stable nanofibrous plume.

**FIGURE 7:**
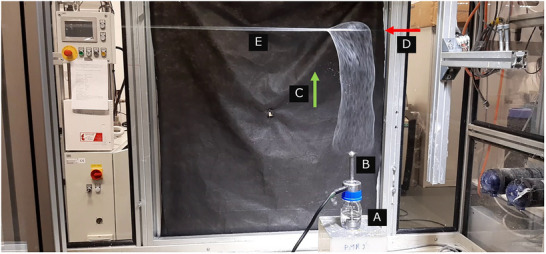
Custom AC electrospinning setup consisting of a polymer solution reservoir (A) feeding a rod‐shaped spinneret (B). The process generates an upward‐directed nanofiber plume (C, green arrow indicates the direction of movement), which is continuously deposited onto a moving PA6 core yarn, not visible due to its small diameter (D, red arrow indicates the direction of movement). At the point of contact, the fibers form a uniform coating, producing a composite nanofibrous yarn (E).

Upon applying an electric potential to the spinneret, a dense plume of nanofibers was generated as a result of the alternating charge polarity (Figure 7). A sinusoidal AC voltage was supplied using a KGUG 36 high‐voltage transformer (Asea Brown Boveri, Switzerland) with a conversion ratio of 36 000 V to 230 V. The voltage input was regulated using an ESS 104 porevariable autotransformer (Thalheimer Transformatorenwerke GmbH, Germany), allowing for a controlled output of 0–250 V from a 230 V input. For all experiments, the effective voltage was maintained at 40 kV with a frequency of 50 Hz.

The resulting nanofiber plume was directed vertically by the action of electric wind and continuously deposited onto a moving PA6 core yarn (Figure 7). The preloaded core of the yarn unwinds from the master bobbin, passes through the eccentric hole of the first twister, and then twists and loops into the shape of a balloon. Thanks to the local twisting and ballooning of the core yarn with its circumferential velocity to be slightly higher than the velocity of the nanofibrous plume, the core is tightly enveloped by nanofibers at the area of contact of the core with the plume of nanofibers as introduced by Valtera et al. [6]. The yarn was drawn at controlled velocities of 10, 15, 20, 25, and 30 m/min and simultaneously twisted to enable uniform nanofiber wrapping. This was achieved using two twirling devices positioned before and after the spinning zone. These devices rotated in the same direction at speeds of 6 000 RPM (inlet twister) and 5 000 RPM (outlet twister), inducing local axial rotation and general ballooning of the yarn to facilitate uniform nanofiber deposition and sufficient adhesion of the nanofibrous envelope to the core yarn.

After fiber deposition, the yarn passed through two sequential drying zones (total length: 2.2 m) to ensure complete solvent evaporation. Each drying chamber had an inner hole with diameter of 3 cm surrounded by cylindrical ceramic wall with controlled heating equipment. The drying temperature was maintained at 40°C using a resistance heating inside the chamber and pre‐heated air provided by Mistral 6 hot air blower (Leister Technologies AG, Switzerland). Finally, the fully formed and compacted composite nanofibrous yarns were wound on an output bobbin.

### 4.2 Release of Chlorhexidine

To quantify the long‐term release of CHX from composite nanofibrous yarns, the UV‐Vis spectroscopy was used. Composite nanofibrous yarns one meter long (*n* = 6) were separately inserted into the wells with 750 µL of phosphate buffered saline (PBS, pH 7.4) in the 48 well plate, in sterile conditions. Yarns were incubated in 5% CO_2_ at 37°C for 1, 3, 7, 14, 22, and 28 days. Each experimental day the PBS was aspirated and fresh 750 µL of PBS was added into the wells. 200 µL of the aspirate, as well as the calibration curve solutions, was added into the UV‐Vis well plates (cat. no. 8404, Thermo Fisher Scientific). The absorbance at 255 nm and 257 nm was detected (Instrument SPARK; Tecan, Switzerland).

### 4.3 Morphological Analysis of as‐Fabricated Yarns

The morphology of the materials was analyzed by scanning electron microscopy (SEM). A Vega 3 scanning electron microscope (TESCAN, Czech Republic) was used for this analysis, and the samples were observed at different magnifications (1 000×, 3 000×, and 5 000×). The accelerating voltage was set to 15 kV. The samples were prepared by gluing small cuttings of material onto a metal target covered with double‐sided adhesive tape. Samples were sputter coated with a 10 nm layer of gold using a Quorum Q150R S Plus sputter coater (Quorum Technologies, Great Britain).

Fiber diameter measurements were performed using ImageJ software (National Institutes of Health, USA). A total of 200 fiber diameters were analyzed from five SEM images (5 000× magnification). For each image, 40 measurements were taken along a defined diagonal to ensure uniformity.

The curl of nanofibers on the surface of composite nanofibrous yarn is described here by stereological estimates of the average curvature and the average torsion values. These quantities are determined using a method proposed by De Hoff et al. [21]. The following applies to the torsion estimator $\left[\underline{\tau }\right]$

(4) $$\left[\underline{\tau }\right]=\frac{\pi }{2}\frac{I_{A}}{N_{L}}$$

where *I_A_* is the number of inflection points per unit area of the projection. The parameter *N_L_* represents the number of intersections between a uniformly random and isotropic test line and nanofibers that occur per unitary length of the test line. The average curvature estimator is given by the relation

(5) $$\left[\underline{k}\right]=\frac{\pi }{2}\frac{T_{A}}{N_{L}}$$

where the symbol *T_A_* indicates the number of tangent positions of a test line per unit area of the nanofibrous material projection. To determine the number of these tangent positions, the test line moves across the projection so that its direction is parallel to the original direction at each position. We refine the estimation of *T_A_* values by averaging over isotropic orientations of this original direction. The analysis of curvature and torsion of nanofibers can be interpreted using a model of a perfect helix, the radius of which is denoted as *a* and the pitch as *2πb*. The relations between the curvature and torsion of the helix and its diameter and pitch are

(6) $$b=\frac{\tau }{k^{2}+\tau^{2}}\;,\mathrm{and}\;a=\frac{k}{k^{2}+\tau^{2}}\;$$

The shapes of helixes having the same values as nanofibers on the surface of composite nanofibrous yarns are depicted using Wolfram Mathematica software.

The number of nanofiber fibrils on a cross‐section of composite nanofibrous yarn was determined using a selected representative sample. To estimate the number of fibrils as isolated parts of an object in the unit area of 2D space, we use an exclusion line test system [47]. The probe of this system is 2D in the shape of a rectangle. Its area is denoted by S(A). The exclusion line is then an infinite, continuous, double‐bent line passing through a part of the boundary of probe A, namely two adjacent sides of the rectangle.

### 4.4 Linear Density of Composite Nanofibrous Yarn

The linear density of the composite nanofibrous yarns (expressed in dtex) was determined using a gravimetric method. A one‐meter length of each sample was weighed using an analytical balance, and the dtex value was calculated using the following formula: Linear density (dtex) = (mass of the 1 m yarn (g) / (length of the yarn (m)) × 10 000.

### 4.5 Mechanical Testing

The PA6 core and PCL_10, PCL_30, PCL+CHX_10 and PCL+CHX_30 composite nanofibrous yarns were evaluated using a straight‐pull test and a knot‐pull test. For straight pull test, yarns one meter in length were placed into Eppendorf tubes with PBS. For the knot‐pull test, the yarns one meter in length were tied into a square knot around a 6 mm‐diameter bar. After removing the bar, the yarns were placed into Eppendorf tubes with PBS. After 30 h the measurement was performed. Ends of the yarns with knot were placed around the grips, the knot was positioned at the center of the gauge length. Tensile properties of the composite nanofibrous yarns were evaluated using a LabTest 6.0051 universal testing machine (LaborTech, Czech Republic) equipped with manual capstan grips and a 10 N load cell (AST, Germany). The load cell complied with EN ISO 7500–1, providing accuracy class 1 from 0.030 N and class 0.5 from 0.1 N. Force and elongation data were recorded at a sampling frequency of 50 Hz. Each test was conducted with an initial gauge length of 250 mm, a crosshead speed of 250 mm·min^−^^1^, and a preload of 10 cN to ensure uniform yarn alignment and tension prior to loading. All measurements were performed under controlled laboratory conditions (22 ± 2°C, 50% ± 5% relative humidity) and took around 30 s. For each yarn type, the average tensile strength, Young's modulus, elongation at break and knot pull strength were calculated as the mean values from ten independent specimens. Because the nanofibrous envelope contributes minimally to load bearing but increases yarn diameter, stress values were normalized using the linear density (dtex) of the PA6 core rather than the overall composite diameter.

### 4.6 Accelerated Enzymatic Degradation of Nanofibrous Envelope

The samples, one‐meter length, were weighed and placed in 48‐well culture plates. The number of samples per test day was three (*n* = 3) and two negative controls (*n* = 2) incubated without the lipase. Testing was conducted over a period of 5 days. Samples were incubated in an enzyme medium consisting of PBS with the addition of sodium azide (0.02%) (Sigma‐Aldrich, Germany) and lipase (Sigma‐Aldrich, Germany) at a concentration of 2.5 U/mL. The total volume of the medium was 1 mL. Negative controls were wetted in PBS only, and served as a reference to compare surface and weight changes for degraded samples. Samples were placed in a humidified shaker (37°C, 50 RPM) throughout the experiment. Culture plates were wrapped with parafilm to prevent evaporation of the medium. Sampling and medium changes were performed every 24 h. Collection was followed by rinsing in distilled water (twice), placing degraded samples on parafilm and drying in a drying chamber at 25°C for 48 h. The dried samples were then used for weight loss and morphological change analysis. After proper drying samples were immediately weighed using a digital balance (PA224C four‐range analytical balance, Ohaus, Switzerland). To determine the percentage weight loss, weights of samples before and after degradation were determined and then subjected to calculation by Equation (7).

(7) $$w_{loss}\;\left[\%\right]=\frac{w_{after}-w_{before}}{w_{before}}\cdot 100$$

where w_before_ is the dry weight [g] before degradation and w_after_ is the dry weight [g] after degradation.

### 4.7 Protein Adsorption and SDS‐PAGE

Bovine Serum Albumin (BSA, Sigma Aldrich, Germany) was used as a model protein to detect adsorption on yarns. The samples, one‐meter length (*n* = 5), were weighed and placed in a 1.5 mL Eppendorf tube. The yarns were incubated with a BSA protein solution (30 mg/mL in PBS). Negative controls were incubated in PBS only. The samples were incubated for 1 h at 37°C. Subsequently, the samples were washed twice in PBS, first wash (W1) and second wash (W2). The samples were then transferred to a desorption solution (D), 1% sodium dodecyl sulfate (SDS, Sigma Aldrich, Germany) in PBS. Samples were incubated for 1 h at RT. Analysis of individual solutions (first wash (W1), second wash (W2) and desorption (D)) was performed using the Pierce BCA Protein Assay Kit (Thermo Fisher Scientific) according to the manufacturer's manual. The absorbance at 562 nm was detected (Instrument SPARK; Tecan, Switzerland).

Protein samples W1, W2 and D were further analyzed by sodium dodecyl sulfate polyacrylamide gel electrophoresis (SDS‐PAGE). The samples were mixed with a sample loading buffer (containing SDS and β‐mercaptoethanol, Merck, Czech Republic) in a 1:1 ratio and heated for 15 min at 95°C. The samples were then centrifuged at 5 000 RPM. For SDS‐PAGE a 10% resolving gel and a 5% stacking gel were prepared for sample separation, 15 µL of samples and 5 µL of protein marker (Precision Plus Protein All Blue Prestained Protein Standards, Bio‐Rad, USA) were loaded into gel wells. The gels were run at constant voltage of 90 V for 90 min. The separation gels were subsequently stained with Coomassie Brilliant Blue R250 staining solution (CCB, Merck, Czech Republic) and left on a shaker with constant stirring for 24 h. The gels were then washed several times in a destaining solution (Methanol, Acetic Acid, distilled water) to completely remove the staining agent. The gels were imaged and analyzed using the GelDoc Go Imaging documentation system (Bio‐Rad Laboratories).

### 4.8 Cell Seeding on Non‐Functionalized Yarns

The yarns were sterilized using ethylene oxide (Anprolene AN74i, H W Andersen Products Ltd, Essex, UK). The non‐functionalized composite nanofibrous yarns and PA6 core yarn, 50 mm in length, were placed into 500 µL tubes. After that, the yarns (*n* = 6) were seeded with 11×10^4^ NIH‐3T3 mouse fibroblasts (LGC Standards, Poland) in 200 µL of growth medium consisting of Dulbecco's Modified Eagle's Medium (DMEM, Merck), 10% fetal bovine serum (FBS, Gibco) and 1% antibiotics (Penicillin‐Streptomycin‐Amphotericin B, Lonza) and were let to adhere for 2 h. Afterwards, the yarns were placed into the well plates and cultured in growth medium, 1 mL per 50 mm length yarn. Cells were cultured in 5% CO_2_ at 37°C for 7 days.

### 4.9 Metabolic Activity Measurement

The metabolic activity of cells was measured using an MTS assay (CellTiter 96 AQueous One Solution Cell Proliferation Assay; Promega, Madison, WI, USA), according to the manufacturer's manual. The absorbance at 490 nm and 690 nm was detected (Instrument SPARK; Tecan, Switzerland).

### 4.10 DNA Quantification

After metabolic activity measurement, yarns seeded with cells were then transferred to the 150 µL of lysis buffer (0.0004% v/v Triton X‐100, 10 mM Tris (pH 7.0), and 1 mM EDTA). After three freeze‐thaw cycles, with vortexing between each cycle, the amount of DNA was measured using the Quant‐iT dsDNA Assay Kit (Life Technologies, Eugene, OR, USA) according to the manufacturer's manual at λ_ex_ = 485 nm, λ_em_ = 523 nm using Instrument SPARK (Tecan, Switzerland).

### 4.11 Confocal Microscopy

Composite nanofibrous yarns and PA6 core yarn seeded with cells were washed in PBS and fixed with frozen methyl alcohol (‐20°C). Afterwards, 3,3′‐diethyloxacarbocyanine iodide (DiOC6(3), Invitrogen, 20 mM diluted 1 000× in PBS) was added for 30 min to visualize the intracellular membranes. Subsequently, the cell nuclei were stained using propidium iodide (PI; 5 µg/mL in PBS, 10 min). Afterwards, yarns were washed three times with PBS, and visualized using confocal microscopy (ZEISS LSM 5 DUO, PI: λ_ex_ = 561 nm, λ_em_ = 630–700 nm; DiOC6(3): λ_ex_ = 488 nm, λ_em_ = 505–550 nm).

### 4.12 Scanning Electron Microscopy of Cell Seeded Yarns

Composite nanofibrous yarns and PA6 core yarn seeded with cells were washed in PBS and fixed with 2.5% glutaraldehyde for 2 h at 4°C. Afterwards, the yarns were washed in PBS and dehydrated in ethanol ranging from 35%‐absolute ethanol and then were let to dry. Yarns were analyzed as described above by Tescan Vega 3 (Brno, Czech Republic).

### 4.13 Statistical Analysis

Quantitative data are presented as mean ± standard deviation (SD). Statistical analyses were performed using GraphPad Prism 9.1.1. Normality of the data was assessed using the Shapiro–Wilk test. For normally distributed data, differences between groups were evaluated by one‐way ANOVA followed by Tukey's post‐hoc test. For non‐normally distributed data, differences were assessed using the Kruskal–Wallis test followed by Dunn's multiple comparison test. Differences were considered statistically significant at *p* < 0.05.

## Funding

This study was supported by the Ministry of Health of the Czech Republic, grant no. NW24‐08‐00133, Student Grant Competition, Technical University of Liberec, grant no. SGS‐2021‐4007 and by the “Centre of Excellence in Regenerative Medicine” project, registration number CZ.02.01.01/00/22_008/0004562, of the European Union Programme entitled Johannes Amos Comenius Operational Program in the call “Excellent Research”.

## Conflicts of Interest

The authors declare no conflicts of interest.

## Supporting information




**Supporting File**: mabi70153‐sup‐0001‐SuppMat.docx.

## Data Availability

The raw and processed data required to reproduce these results are available by contacting the corresponding author.
