# Supplementary material for: Antimicrobial Functionalization of Composite Nanofibrous Yarns as Surgical Sutures
Source: Macromol Biosci. 2026 Feb 24;26(2):e00510. doi: 10.1002/mabi.202500510 (PMC12931029; doi:10.1002/mabi.202500510)
Supplement: Supplementary file 1 — Supporting File: mabi70153‐sup‐0001‐SuppMat.docx. [file MABI-26-e00510-s001.docx]

Supporting Information

Antimicrobial Functionalization of Composite Nanofibrous Yarns as Surgical Sutures

Věra Hedvičáková^*^, Manikandan Sivan, Divyabharathi Madheswaran, Kristýna Havlíčková, Šárka Hauzerová, Maxim Lisnenko, Jan Valtera, Jaroslav Mikule, Kateřina Strnadová, Věra Jenčová, Eva Kuželová Košťáková, David Lukáš

Table of Contents

S1. Calculation of Fiber Packing Density

S2. Calculation of Porosity

S3. Calculation of Mean Pore Size

Abbreviations (for Sections S1-S3)

S4. Fitting of CHX Release Profiles to First-Order and Bi-Exponential Kinetic Models

Figures S1–S4

References

S1. Calculation of Fiber Packing Density

For the volume of nanofibrous material:

*V*_FN_=π*D*^2^ *L*_NF_. Equation (S1)

The length of nanofibers in the sheath is given by:

$L_{NF}=\frac{V_{NF}}{\pi D^{2}}$. Equation (S2)

The nanofiber volume *V*_FN_ can be calculated from the linear mass of the nanofibrous sheath:

$V_{NF}=\frac{L_{NF} L}{\varrho}$, Equation (S3)

where the product of *the quantities L_NF_ and L* has the meaning of the mass of the nanofiber material in the nanofiber envelope. The total volume of the nanofiber sheath V_S_ made of nanofibers including pores is calculated using the inner and outer radii of the nanofiber envelope, which we find out from SEM images of composite nanofiber yarns

$V_{S}=\pi\left( \frac{d_{2}^{2}}{4}-\frac{d_{1}^{2}}{4} \right)L=\frac{\pi}{4}\left( d_{2}^{2}-d_{1}^{2} \right)L.$ Equation (S4)

For fiber packing density *Z_NF_* of the nanofiber envelope*,* we get the relation

$Z_{NF}=\frac{V_{NF}}{V_{S}}=\frac{L_{NF} L}{\varrho}\frac{4}{\pi\left( d_{2}^{2}-d_{1}^{2} \right)L}=\frac{4}{\pi}\frac{L_{NF}}{\varrho\left( d_{2}^{2}-d_{1}^{2} \right)}$. Equation (S5)

S2. Calculation of Porosity

Porosity φ is defined as:

$\phi=1-Z_{NF}$. Equation (S6)

Conversion of tex (g/km) to SI units (kg/m):

$\frac{1g}{\mathrm{km}}=\frac{{10}^{-3}\mathrm{kg}}{{10}^{3}m}={10}^{-6}\frac{\mathrm{kg}}{m}$.

Density of PCL at 20 °C: ρ = 1.145 × 10³ kg/m³.

S3. Calculation of Pore Size Distribution

Determination of the pore size distribution is based on the method described by Masounave et al.^[1]^
The fiber length density *L*_NF_/*V*_S_ is determined using the stereological estimator introduced by Cruz-Orive. ^[2]^

$\frac{L_{NF}}{V_{S}}=\frac{2Q}{S}=2N_{S}$. Equation (S7)

This relation applies to a homogeneous and isotropic fibrous system. From this, the expression for the numerical surface density of nanofiber cross-sections can be readily derived:

$N_{S}=\frac{1}{2}\frac{L_{NF}}{V_{S}}$. Equation (S8)

The volume of the *V_S_* of the nanofiber envelope can be determined from SEM images of perpendicular sections of composite nanofiber threads using Equation (S4). The length of nanofibers in the nanofiber envelope can be found from the Equation (S2), $L_{NF}=\frac{V_{NF}}{\pi D^{2}}$. After substituting from Equation (S2) for *L_NF_*, we get

$N_{S}=\frac{1}{{2V}_{S}}\frac{V_{NF}}{\pi D^{2}}$. Equation (S9)

For this relation, we must determine the volume of nanofibrous material in the *V*_NF_ nanofiber sheath from the linear sheath of the nanofiber sheath, which allows us to use the Equation (S3). After substitution into the Equation (S9), we obtain$V_{NF}=\frac{L_{NF} L}{\varrho}$

$N_{S}=\frac{1}{{2V}_{S}}\frac{1}{\pi D^{2}}\frac{L_{NF} L}{\varrho}$. Equation (S10)

The last modification is to substitute in the previous equation after *V_S_* from the Equation (S4). So we get $V_{S}=\frac{\pi}{4}\left( d_{2}^{2}-d_{1}^{2} \right)L$

$\boldsymbol{N}_{\boldsymbol{S}}=\frac{L}{2\pi D^{2}\varrho}\frac{4}{\pi\left( d_{2}^{2}-d_{1}^{2} \right)L}=\frac{\boldsymbol{2}\boldsymbol{L}_{\boldsymbol{NF}}}{\boldsymbol{\pi}^{\boldsymbol{2}}\boldsymbol{\varrho}\boldsymbol{D}^{\boldsymbol{2}}\left( \boldsymbol{d}_{\boldsymbol{2}}^{\boldsymbol{2}}\boldsymbol{-}\boldsymbol{d}_{\boldsymbol{1}}^{\boldsymbol{2}} \right)}$. Equation (S11)

The probability density function of pore radii is given by Masounave et al.^[1]^:

$f\left( r \right)=2\pi N_{S}re^{-\pi N_{S}r^{2}}$. Equation (S12)

The most probable pore radius $r_{p}$ satisfies d*f*(r)/d*r* = 0, leading to:

$\frac{df\left( r \right)}{dr}=2\pi N_{S}e^{-\pi N_{S}r^{2}}-\left( 2\pi N_{S}r \right)^{2}e^{-\pi N_{S}r^{2}}=0$.

After truncating this relation with a non-zero expression, we get ${2\pi N_{S}e}^{-\pi N_{S}r^{2}}$

$1-2\pi N_{S}r^{2}=0$

The most probable pore radius $r_{p}$ is expresed as:

$r_{p}=\frac{1}{\sqrt{2\pi N_{S}}}$. Equation (S13a)

For the average pore radius size, the following applies

$r_{s}=\sqrt{\frac{ln2}{\pi N_{S}}}$. Equation (S13b)

Abbreviations (for Sections S1-S3)

*V*_FN_ – Volume of nanofibrous material in the sheath

*V*_S_ – Volume of the nanofibrous sheath including pores

*d_1_ –* Inner radius of the nanofibrous sheath

*d_2_ –* Outer radius of the nanofibrous sheath

*D –* Average fiber diameter

*Ρ* – Density of polymer

*L_Y_* – Length of yarn

*L*_NF_ – Length of nanofibrous material

*S –* Cross-sectional area of the nanofibrous material

*Q* – Number of fiber cross-sections in area S

*N_S_ –* Areal number density of fiber cross-sections (*N_S_ = Q/S*)

*M*_L,tex_ - Linear mass of nanofibrous sheath in tex

*M*_l,,SI_ – Linear mass of nanofibrous sheath in SI units

*Z_NF_* – Volume fraction of nanofibers in the nanofibrous shell

φ – Volumetric fiber packing density of the nanofibrous sheath

*r –* Pore radius

*f*(*r*) – Probability density of pore radius r

S4. Fitting of CHX Release Profiles to First-Order and Bi-Exponential Kinetic Models

S4.1. Methodology

To evaluate the mechanism of chlorhexidine (CHX) release from composite nanofiber yarns, the experimental release profiles were analysed using standard kinetic models. As a first approach, a simple first-order model,

$\frac{dc}{dt}=-kc=-\frac{c}{\tau}$ , Equation (S14)

was applied, where *c*(*t*) is the concentration of CHX in the release medium, *k* is the first-order rate constant, and $\tau=\frac{1}{k}$ is the characteristic (relaxation) time. The analytical solution of Equation (S14) is:

$c=A \exp\left( -\frac{t}{\tau} \right)$, Equation (S15)

which is typically suitable for water-soluble drugs released from porous matrices.^[3]^ However, Equation (S15) did not provide a satisfactory regression for CHX release from the composite nanofiber yarns investigated in this study. The poor agreement indicates that the release process is governed by at least two distinct mechanisms—most plausibly a fast release of CHX located at or near the fiber/yarn surface and a slower release of CHX embedded deeper within the fiber/yarn volume. Such behaviour is commonly approximated by a bi-exponential model:

$c=A \exp\left( -\frac{t}{\tau} \right)+B \exp\left( -\frac{t}{T} \right)$, Equation (S16)

where τ and $T$ are so called short and long relaxation times, respectively.

S4.2. Discussion

Application of Equation (S16) led to a robust fit for all samples. The short relaxation time was consistently approximately **1 day**, independent of the production (winding) speed. In contrast, the long relaxation time increased systematically with production speed, ranging from **24 to 35 days.** These two time constants quantitatively support the dual-mechanism hypothesis involving rapid surface release and slow diffusion-controlled release from the fiber interior.

The fitted relaxation times further support the hypothesis of two distinct release mechanisms. The **short relaxation time** *τ* remains nearly constant across all production speeds, varying only within **approximately 5–8%**. This weak dependence on the winding speed indicates that the fast-release mechanism—associated with superficially located or weakly bound CHX—is largely unaffected by changes in yarn production parameters. This is consistent with the expectation that nanofibers produced by AC electrospinning exhibit essentially identical fiber morphology regardless of the winding speed.

In contrast, the **long relaxation time** *T* shows a **substantially larger variation of approximately 35–45%,** increasing systematically with winding speed. This trend suggests that structural changes occurring at the yarn level—not at the level of individual fibers—govern the slow-release process. At higher winding speeds, the nanofiber sheath is likely compacted more tightly around the core filament, creating a **less permeable diffusion pathway** for CHX molecules located within the yarn bulk. The reduced effective porosity or increased packing density of the nanofiber envelope would therefore prolong the diffusion-controlled release phase.

This interpretation is consistent with our stereological measurements, which showed no significant changes in individual fiber morphology but did reveal differences in the spatial organization and packing of nanofibers within the yarn envelope. These structural variations at the yarn scale provide a plausible mechanistic explanation for the observed dependence of the long relaxation time on production speed.

Figures


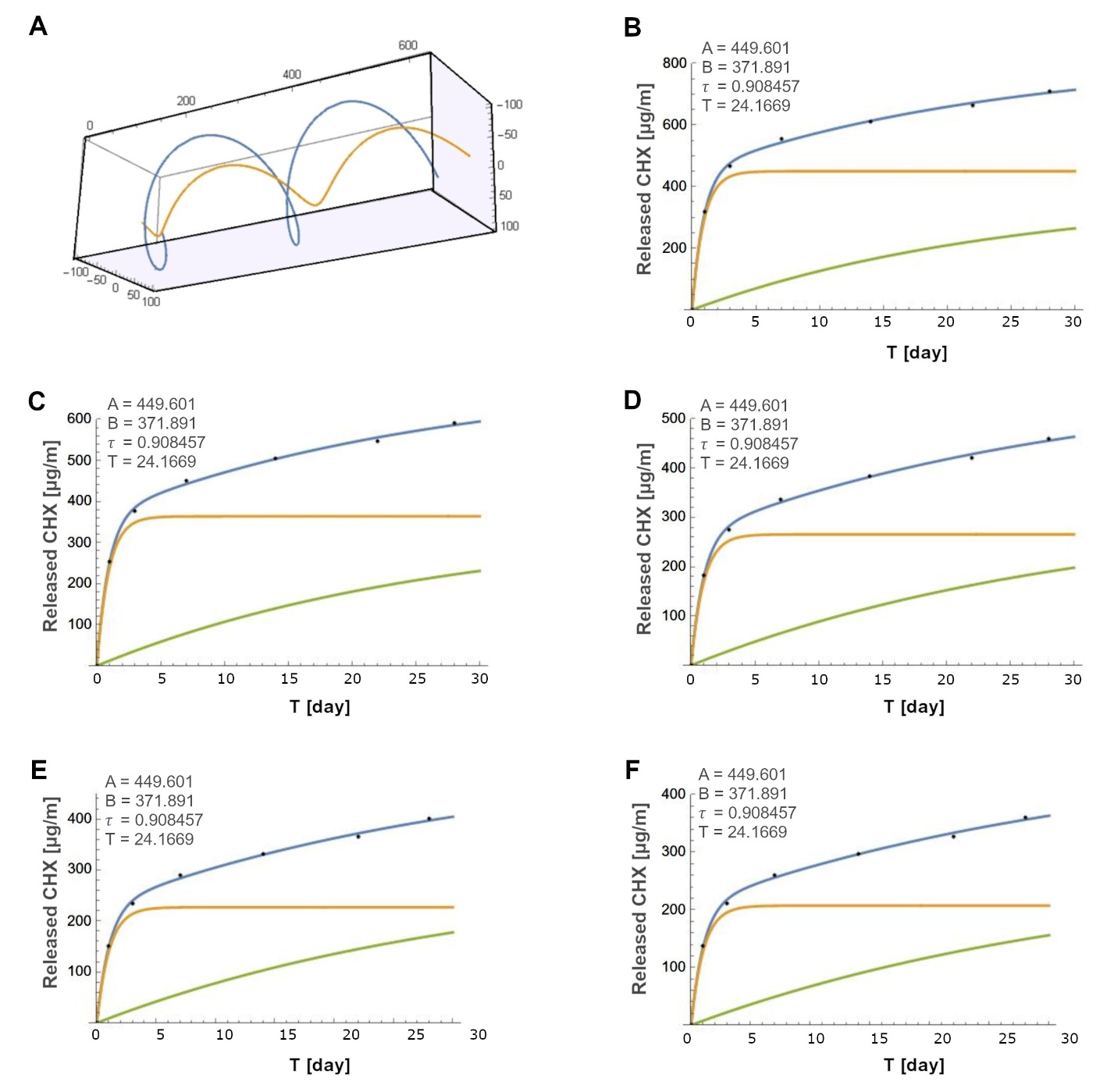


**Figure S1:** A) Helices representing curvature c = 0.0101 μm^-2^ and torsion τ = 0.0109 μm^-2^ of surface nanofibers of composite nanofibrous yarn PCL_10 (orange). Helices representing curvature c = 0.00835 μm^-2^ and torsion τ = 0.00353 μm^-2^ of surface nanofibers of composite nanofibrous yarns PCL_30 (blue). The coordinate axes in the figure are plotted in μm. Figures B) PCL_15, C) PCL_15, D) PCL_20, E) PCL_25, F) PCL_30 show shorter (𝜏, orange) and longer (*T,* green) relaxation times together with cumulative release of chlorhexidine (blue).


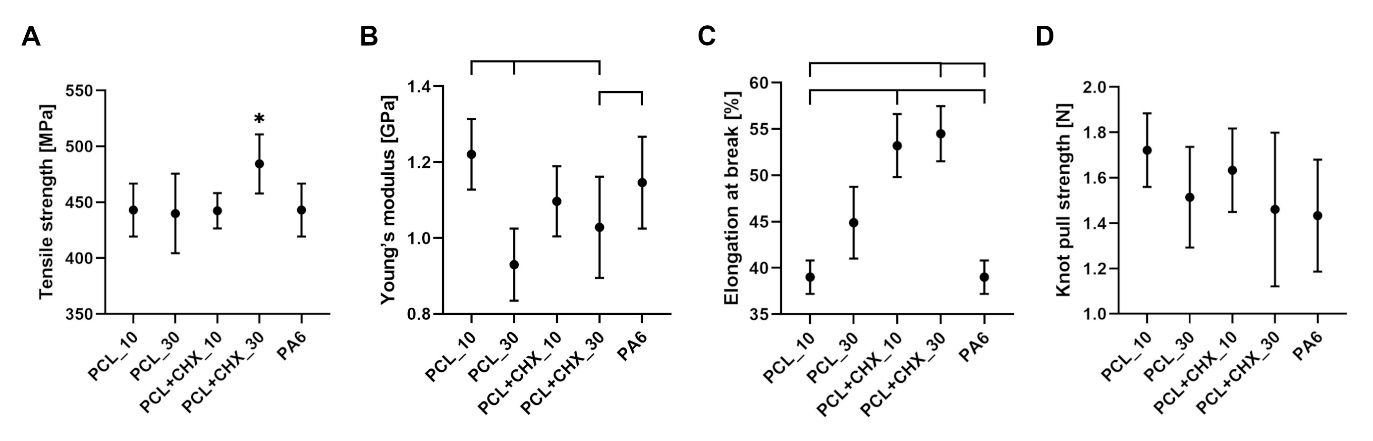


**Figure S2:** Mechanical properties of the tested materials. (A) Tensile strength, (B) Young’s modulus, (C) elongation at break, and (D) knot pull strength of PCL_10, PCL_30, PCL+CHX_10, PCL+CHX_30 composite nanaofibrous yarns and PA6 core yarn. Data are presented as mean ± SD. Statistical significance is denoted above the columns (p < 0.05), * means the statistically highest value on the tested day.

**
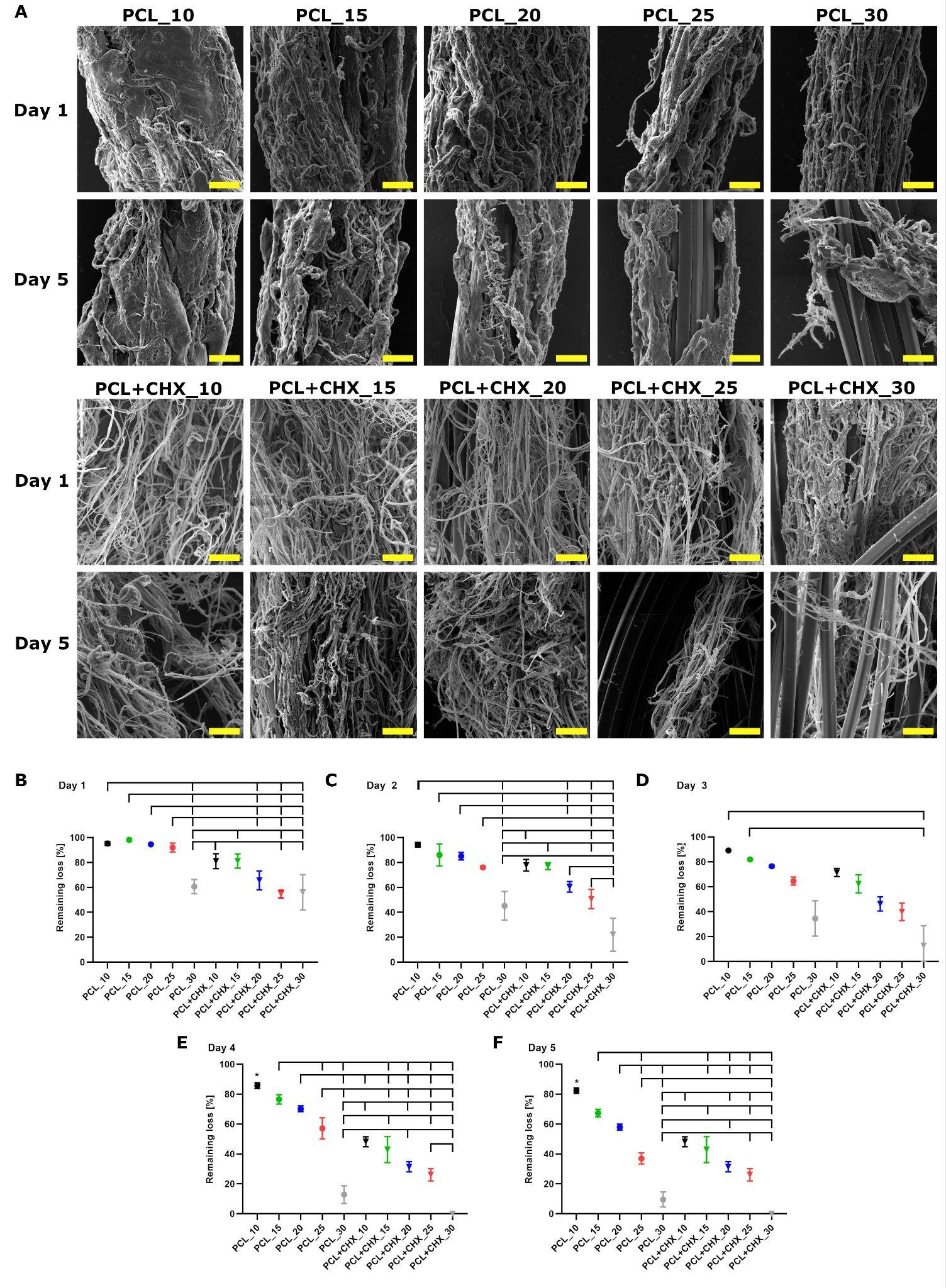
**

**Figure S3**: A) SEM images of pristine and CHX-loaded composite nanofibrous yarns after 1 and 5 days of degradation. The exception is PCL+CHX_30 sample, the envelope degraded after 3 days. Scale bar 50 µm. Statistical significance of degradation on days B) 1, C) 2, D) 3, E) 4 and F) 5. Statistical significance is denoted above the columns (p < 0.05), * means the statistically highest value.


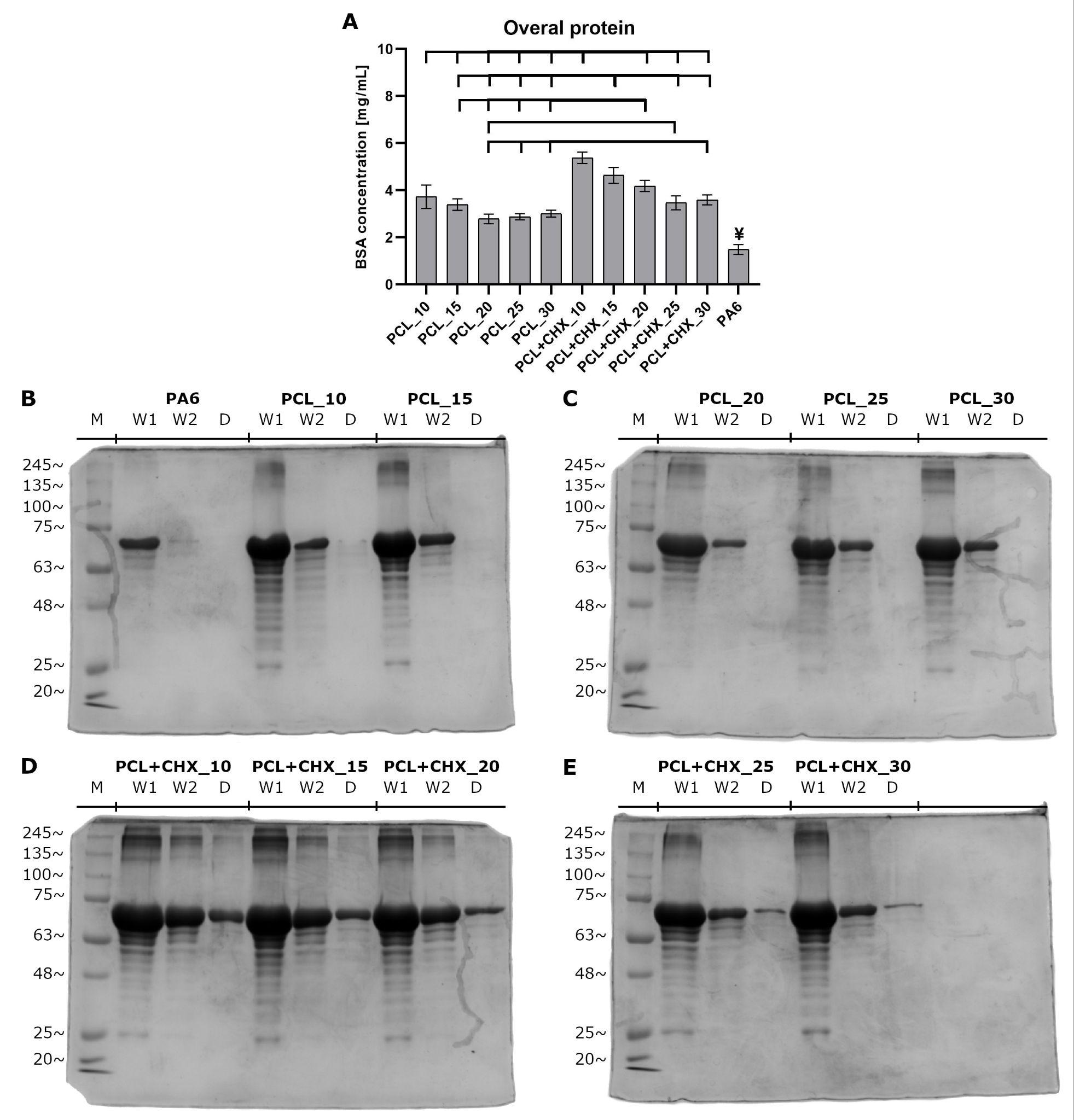
**Figure S4:** A) Summary graph showing overall proteins (capillary-, weakly- and strongly bound proteins) adsorbed on the composite nanofibrous yarns. SDS-PAGE analysis of adsorbed proteins on samples B) PA6, PCL_10, PCL_15, C) PCL_20, PCL_25, PCL_30, D) PCL+CHX_10, PCL+CHX_15, PCL+CHX_20, E) PCL+CHX_25, PCL+CHX_30. Samples were separated in a polyacrylamide gel containing capillary-bound (W1), weakly bound (W2) and strongly bound (D) proteins. Marker (M) is included in each gel. Statistical significance is denoted above the columns (p < 0.05), ¥ means the statistically lowest value.

References

1. Prediction of permeability of non‐woven geotextiles from morphometry analysis - Masounave - 1981 - Journal of Microscopy - Wiley Online Library. Accessed November 29, 2025. https://onlinelibrary.wiley.com/doi/abs/10.1111/j.1365-2818.1981.tb01202.x
2. Cruz-Orive LM. Stereology of single objects. *Journal of Microscopy*. 1997;186(2):93-107. doi:10.1046/j.1365-2818.1997.1380695.x
3. Dash S, Murthy PN, Nath L, Chowdhury P. Kinetic modeling on drug release from controlled drug delivery systems. *Acta Pol Pharm*. 2010;67(3):217-223.
